# COMMUNICATION OF DEATH AND GRIEF SUPPORT TO THE WOMEN WHO HAVE LOST A NEWBORN CHILD

**DOI:** 10.1590/1984-0462/;2018;36;4;00013

**Published:** 2018

**Authors:** Marina Uchoa Lopes Pereira, Laura Lamas Martins Gonçalves, Cristina Maria Douat Loyola, Patrícia Sampaio da Anunciação, Rosane da Silva Dias, Irla Nunes Reis, Lays Amorim Silva Pereira, Zeni Carvalho Lamy

**Affiliations:** a Universidade Federal do Maranhão, São Luís, MA, Brasil.; b Universidade CEUMA, São Luís, MA, Brasil.

**Keywords:** Perinatal death, Bereavement, Social support, Humanization of assistance, Morte perinatal, Luto, Apoio social, Humanização da assistência

## Abstract

**Objective:**

To analyze the communication of a child’s death and the grief support provided to the women during puerperium.

**Methods:**

This is a qualitative study performed at a capital of the Northeast region of Brazil. Semi-structured interviews were carried out with 15 women, whose children died from July 2012 to July 2014. The interviews contained questions about the child’s death and the grieving process. The content analysis was performed with a thematic approach.

**Results:**

The women expressed the suffering and the anguish of the loss of a child, sometimes aggravated by the way in which the news of death was delivered, and by the lack of support offered in the coping process. Two empirical categories were found: receiving the news of death and going back home empty-handed. The health care teams are not prepared to deliver bad news, nor to give support to women who lose a newborn child. According to the women, the support received from the family and religion helped them in the grieving process.

**Conclusions:**

The results indicate the need for professional qualification for the delivery of bad news and for grief support. They also showed the need for institutional policies that offer support to the professionals. Besides, the articulation with the primary health care team is imperative for the continuity of care.

## RESUMO

**Objetivo:**

Analisar a comunicação da morte do filho e o apoio ao luto de mulheres no período puerperal.

**Métodos:**

Trata-se de uma pesquisa com abordagem qualitativa realizada em uma capital do Nordeste. Foram aplicadas entrevistas semiestruturadas com 15 mulheres cujos recém-nascidos faleceram entre julho de 2012 e julho de 2014. As entrevistas abordaram questões acerca da morte do filho e do processo de luto. Foi realizada Análise de Conteúdo na modalidade temática.

**Resultados:**

As mulheres expressaram o sofrimento e a angústia diante da perda do filho, algumas vezes agravados pela forma da comunicação da notícia e pela falta de suporte ofertado para o enfrentamento. Foram encontradas duas categorias empíricas: receber a notícia da morte e voltar para casa de mãos vazias. As equipes não estão preparadas para a comunicação de notícias difíceis nem para o suporte às mulheres que perderam filhos recém-nascidos. Para as mulheres, o apoio recebido pela família e pela religião ajudou no processo de luto.

**Conclusões:**

Os resultados indicam a necessidade de capacitação profissional para comunicação de notícias difíceis e suporte ao luto, bem como a formulação de políticas institucionais que apoiem e ofereçam cuidado aos trabalhadores. Além disso, é necessária a articulação com as equipes da atenção básica para a continuidade do cuidado.

## INTRODUCTION

The loss of a child is an extremely painful experience, which exposes human beings to their own impotence.^1^ This matter becomes even clearer when such a loss occurs in the neonatal period, since it implies a very specific type of grief, one that is slow and painful, involving individual aspects of the parents and their relationship dynamics to face this situation.^2^ Parents who go through this experience live a moment of crisis and need to adjust to the new situation,^3^ and it is essential that they be free to live and express their pain and grief^4^, and that they receive support from the health team accompanying them.^5^

It can be even harder for the mother, especially because of the physical and psychological experience of pregnancy and hormonal changes.^6^ The grieving process involves adjusting to the loss, which means suffering, as well as the ability to find some hope, comfort, and alternatives of life.^7^ People in grief look for meaning in that transition, not only in the personal and family scopes, but also in the social and cultural spheres, so grief has a social role.^8^ ^,^ ^9^

In the case of death of infants, the silence, which is very common among people who are close to the grieving families, may give the feeling that this death is not considered significant; after all, “he” or “she” had not yet been introduced socially.^10^ In this sense, this death is socially invisible, and professionals must be careful not to reproduce this idea.^11^ That is why it is important for health services to provide sensitive care to families who have lost their babies early,^11^ caring for the communication of death and for the provision of support to the woman and the family.

This study aimed at analyzing the communication of a child’s death and grief support to the women in the puerperal period.

## METHOD

This is a qualitative study, which is part of a larger project, carried out with women living in the city of São Luís, who have had deliveries in different maternity wards from July, 2012, to July, 2014. The participants were identified based on the death certificates (DCs) of their newborn children, registered in the Mortality Information System (SIM).

The inclusion criteria were: mother living in the city of São Luís, gestational age equal to or higher than 32 weeks, and weight at birth equal to or higher than 2500 g. These criteria aimed at excluding newborns with higher chances of death, and gestational age of 32 weeks was conditioned to the classification ranges of duration of pregnancy established in the DC. The exclusion criteria were: women with mental impairment, which could mean cognitive impairment.

We found 410 DCs of newborns in the study period, and, based on the inclusion criteria, 55 were selected; of these, nine had no address and were excluded. Based on the addresses, we identified the health sanitary districts of the households, and the coverage status by the Family Health Strategy (ESF) program, thus the initial sample contained 31 women. We contacted the health community agents to locate the household, and to request an authorization for the visit. Of the 31 women, 15 accepted to participate in the study.

The sociodemographic data were extracted from the DCs (place of occurrence and cause of death, history of previous gestational loss, gestational age), and from a structured questionnaire that was previously elaborated, contemplating the following variables from the mother - age, skin color, schooling, occupation, religion, marital status and obstetric history - and the newborn - weight at birth and birth conditions.

The diagnosis of preventable death was obtained in the File of Investigation of Infant and Fetal Death, from the Epidemiological Surveillance Center, in the Municipal Secretary of Health, based on the List of Preventable Deaths by Interventions from the Unified Health System (SUS).^12^

There were semi-structured interviews conducted by the main researcher in the household, on dates and times established by the interviewees, recorded and then transcribed, using a script with open questions, including questions related to the death of the child and the grieving process.

There were five workshops using the technique of Content Analysis, in the thematic modality, according to the steps of the pre-analysis, categorization and interpretation.^13^

This study was approved by the Research Ethics Committee of the University Hospital at Universidade Federal do Maranhão (HU/UFMA). The identities of the women were kept anonymous. Their names were replaced by those of women known nationally and/or internationally for losing their children, or who somehow fought for the rights of women and children.

## RESULTS AND DISCUSSION

We interviewed 15 women, aged between 20 and 32 years, mostly brown skinned. As to schooling years, 7 had 4 to 6; 6, 8 to 11; and 2, 12 or more. Six were housewives, one was a student, and eight had a paid occupation. Eight were married or were in a stable union, six were single, and one was divorced. Only one was in the first pregnancy. Three had already had stillborn children. Nine had had a term pregnancy, eight had vaginal delivery, and two did not undergo prenatal care. Nine women reported some type of problem in pregnancy, but only three were referred to specialized prenatal care. Four babies had malformation, but in only two cases it was identified in prenatal care.

Regarding the deaths, seven occurred on the first day of life, four between one and seven days, and four after the eighth day. Five occurred due to sepsis, three to malformation, three due to respiratory causes, two because of hypoxemia, one due to heart problems, and one because of perinatal conditions. All deaths occurred in maternity wards or reference hospitals of SUS, and 13 were considered as preventable.

The statements of the interviewees about the death and the grieving process express suffering and anguish facing the loss of a child, and were organized in two empirical categories: receiving the news of death and going back home empty-handed.

### Communication of death

Based on the statements, we observed difficulties from the professionals to communicate not only the death, but also the news that something was wrong with the newborn, showing flaws in the communication between professionals and patients.

The women realized something was wrong with their babies, mainly after birth, and especially because of changes such as crying, paleness, change in skin color, changes in breathing and agitation. Many reported delay, from the health team, to pass on information about the clinical status of the baby.

I looked at her way. Because every child who is born cries right after, and she was quiet. And I said: she is not fine. They took her and ran, but didn’t tell me anything. (Zuzu)

It is very important that the professionals be available to inform about the procedures, by creating conditions so that the users can share doubts and yearnings.^5^ ^,^ ^14^

The news of death was mostly addressed by the health team, mainly by nurses and doctors. Some, however, could not tell which professional broke the news. The attitudes of these professionals point to lack of preparation, as well as violence, in the communication of death.

She was rude and didn’t know how to talk: ‘Who is in bed x?’. I said: ‘It’s me’. And she answered: ‘Your son just died. You can prepare the wake’. (Lucinha)

They asked me: ‘Oh, why are you crying?’ What do you mean, why am I crying? What kind of a question is that? (Bertha)

When she said my name, I started to cry, and she said: ‘God, she is already crying, I didn’t even tell her what happened’. Then, she came close to me and said: ‘look, mom, God knows what He is doing’ No! It is not like that! This is what upset me the most, you know? Her saying: ‘look, mom, God took him, but that is just how it is’. No! (Frida)

The lack of sensitivity from the health team was identified at the time of hospital admission, birth and labor care, as well as in the communication of the news. The reports refer disregard and abandonment in several situations. One of the interviewees, for example, told that after losing her child, she stayed at a nursing room with new mothers feeding their babies, without any concern with the singularity of her context. Another situation that shows institutional violence is the report of telling the news of a child’s death on the telephone:

My cell phone rang in the middle of the night. It was them, letting me know that she had died ten minutes ago. That was all... (Anita)

I answered the phone and the woman said: ‘is this the mother of the baby of that day?’ I said ‘yes’ and she answered: ‘mom, I am sorry to inform you, but your baby died. (Tassia)

Montero et al.^15^ showed that health professionals are still unprepared regarding care in situations of perinatal loss. The findings in this study indicate that many end up acting cold, especially due to the lack of strategies and ability to handle the demands presented by the parents; besides, they do not recognize their role in the handling of this traumatic experience.

The communication of the news of death presents itself as a challenge for health professionals, who are often little skilled to handle the pain of another individual;^6^ ^,^ ^16^ when they do, they establish a relationship of affection^17^, instead of empathy, which shows the lack of preparation to communicate difficult news, and to provide emotional support for the parents. This reinforces the importance of training to give difficult news, in a way that the professional is confident enough to fulfill this task^18^ ^,^ ^19^ ^,^ ^20^.

Another situation that can be an important marker of the need for institutional changes in the work process was told by Zilda:

The doctors only came in to give the medication and asked: ‘where is the child?’ (Zilda)

In that case, the child was already dead. The professional care of searching for information before contacting the mother is very important to prevent the worsening of the pain.

The professional approach, and even that of family members, despite having been identified as supportive, was often inadequate, as reported:

‘Mom, it is just like that’. She said I was young, then I answered: ‘Doctor, but you don’t know the pain that I’m feeling, I have been waiting for my daughter for five years, this is not easy’ (Anita)

And she said: ‘Go on, my girl, you will soon have another baby. Wait one year, after that you can get pregnant again’ (Frida)

According to Iaconelli,^21^ it is common for mothers to hear sentences like: “take it easy, you are young and can have other children”; “it was better this way…”. We can infer that one of the reasons for such attitudes is the difficulty that people have in getting in touch with sadness, since nowadays we are experiencing a movement of total suppression of feelings. Facing that loss, many are impelled to go back to their routines as fast as possible, pretending that nothing has happened.^22^ The statements reinforce the idea that death is a social taboo, and the difficulties to deal with this communication seem to affect everyone.

 The way death is communicated has a long-lasting repercussion for the family.^23^ The content and the form of this communication are equally important, considering that the news of death is the beginning of the realization about the loss and the healthy grieving experience.

For the elaboration of grief, people must be encouraged to share the feelings caused by the loss.^6^ ^,^ ^9^ In grief, there is no formula to mitigate the pain, but it is possible to be present and to show the person in grief that he/she is not alone, and that living the loss is necessary.^8^ In this sense, the health professionals need to be much better prepared to provide care and support in these situations.^20^ They need to have technical experience and to embrace an ethical and cozy posture, since this is how the family members will gain trust and safety.^3^ ^,^ ^24^

The effective and affective communication minimizes difficulties and uncertainties, besides strengthening the feeling of safety, which facilitates good relationships, vital for the quality of care, and helps in the understanding and acceptance of death.^9^ ^,^ ^18^ ^,^ ^19^ ^,^ ^25^

Part of the professionals’ preparation is the possibility that they have institutional support to deal with limit situations,^19^ which can generate suffering and increasing levels of sickening. However, unfortunately, institutional policies addressed to the attention and health care of the employees are still insufficient.^18^

### Going back home empty-handed

Going back home without the child was one of the most difficult moments reported by the interviewees. This represented the reality of losing a child, followed by the sensation of emptiness and impossibility to begin a new phase for the family, which would occur with a baby in the house.

The worst part is coming home without the baby. This is bad, it’s what hurts the most [...] getting home, looking at all of the baby’s things [...] (Cissa)

The discharge from the maternity ward can be pictured as a moment of joy, and symbolizes an event of social presentation of the baby to the family this baby is about to enter.^26^ The death of the baby becomes a situation of difficult personal struggle, besides social embarrassment.^10^

The death of a child represents the beginning of a difficult journey. For the elaboration of this loss, the parents need to build a new reality, considering the investment and the expectations as to the future of a child that is no longer there.^27^

Most of the interviewees did not have a chance to participate in the wake of their children. Some justified they were still hospitalized. However, others, who could have lived this experience of the perception of losing a child, as support to elaborate the grief, mentioned they were encouraged not to do it.

I regret it and I wish I could go back to that moment, because I wanted to have had unwrapped my daughter, given her a bath and burried her with clothes, you know? As any other child... But I was in great despair, and they had already cut her, her head was all shaved, she was so ugly that I didn’t have the courage to look. (Anita)

Maybe, if Anita had been encouraged to, she would have been able to face her pain and care for her dead daughter, so now she would not feel this regret. Do health professionals recognize this role as inherent to the care of women who have lost their children?

According to Oishi,^2^ for the experience of grief, it is important to encourage the parents to deal with their children’s death by indicating actions like seeing and touching the dead baby, choosing a name and place of burial, as well as having a wake. However, the team must be sensitive to respect the singularity of the situations.^3^ ^,^ ^11^

The rites of passage are intermediate and temporary moments of imprecision and crisis, enabling the individual to reflect about his or her existence in society. Among them, death-related rituals refer to the wake ceremony, but also to details like: picking up the body, washing the dead body, choosing the clothes and the place for the wake and burial. Their role is to symbolize an experience of loss and separation.^28^

And my mother didn’t want to give the News to the whole family, to the Brothers, because the body didn’t come, you know? So I thought that. But, then, after a while, I was like: ‘Mom, I needed everyone around me’. I had to be with my sisters in this first moment after his death. (Èdith)

Grief is mostly experienced in the family environment, and mutual support helps in the process of adjusting to the loss.^1^ ^,^ ^29^ Even though the family participates in this suffering, the members need to understand that the parents, especially the mother, need unconditional emotional support.^23^

Religion was referred to as an important factor for the acceptance of death. In some reports, death was seen as a “God’s will”.

But we have to understand, right? It’s God’s will, not ours. (Zilda)

God is not obliged to justify anything to us, right? We have to accept it. (Èdith)

Higher levels of religious involvement are positively associated with indicators of psychological well-being, as well as better physical and mental health. Religious beliefs and practices can reduce the feeling of abandonment and loss of control that accompanies the sickening processes, providing support and relieving the pain.^1^ ^,^ ^26^ ^,^ ^30^ The support of family members and religious belief work as protective factors to deal with the pain caused by death.^9^ ^,^ ^23^

These women have told us about their grieving stories, and, for many of them, this was the only chance to narrate the loss of their children, which can contribute with the elaboration or the re-signification of the experience. On the other hand, this scenario led to one of the study limitations, considering that some did not accept to participate, justifying they did not want to revisit this painful experience. Other limitations refer to the difficulty to ensure privacy in the household, so some interviews were interrupted, continuing later, and to the difficulty to locate the women who had changed address.

## CONCLUSION

The conclusion is that many teams are not prepared to communicate difficult news, nor give the support to women who have lost their newborn children, indicating the need for professional training and institutional policies that support and provide care to the workers.

For the interviewed women, two things have helped in the grieving process: the support received from the family and religion. It is important to mention that it is essential that such support be offered also by the health team, especially in the maternity ward and in the first moments after returning home.

Finally, the responsibility of the team does not end after communicating the death. It is necessary to let the mother, the father and the family aware that they can return to the hospital, in case they wish to, to talk about the death and clarify any doubts. This return must be with the same Neonatology team, especially with a professional who has a closer relationship with the family. Besides, it is essential to articulate with the primary care team, so that care can be continued.
